# Supplementary material for: Proteome partitioning constraints in long-term laboratory evolution
Source: Nat Commun. 2024 May 14;15:4087. doi: 10.1038/s41467-024-48447-2 (PMC11094134; doi:10.1038/s41467-024-48447-2)
Supplement: Supplementary file 1 — Supplementary Information [file 41467_2024_48447_MOESM1_ESM.pdf]

Supplementary Information

Mori et al., *Proteome partitioning constraints in long-term laboratory evolution*

Supplementary Figures

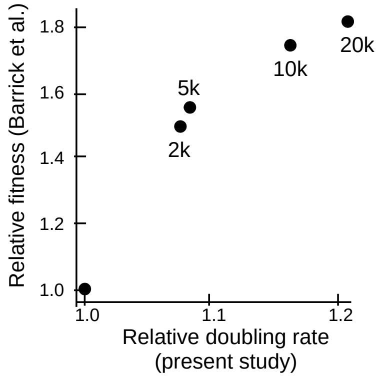

Figure S1 – Comparison between the relative fitness and the exponential growth rate: The relative fitness and the doubling rate increase monotonically increase with generation number. Fitness data is from Barrick *et al.*[1] Growth rate data in Supplementary Data 2.

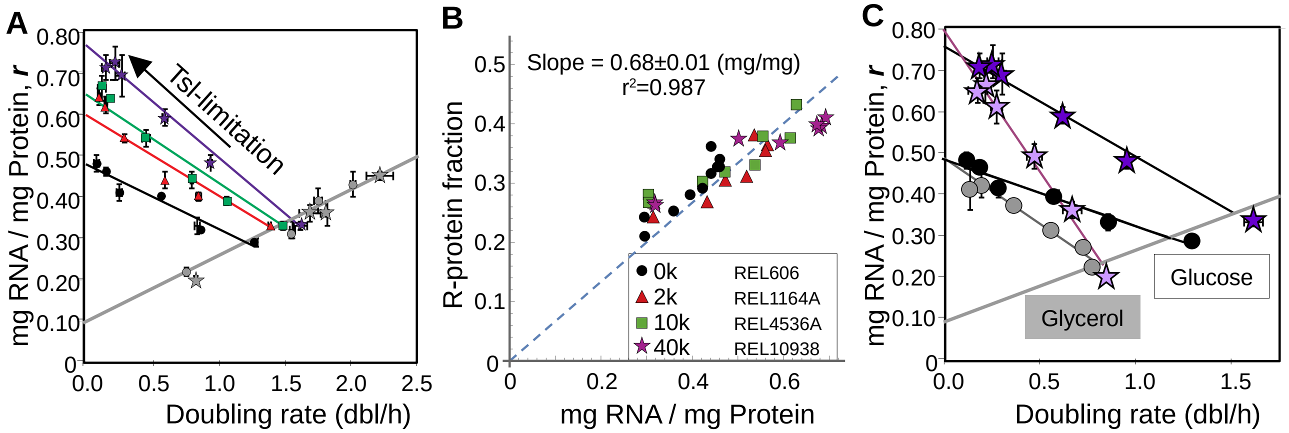

Figure S2 – Ribosome abundance in various nutrient conditions: **A.** Using RNA/protein ratio as a proxy for the ribosome abundance, we observe no change over 40k generations in the abundance under nutrient modulated growth (grey), and we observe the same considerable increase in the maximum intercept under translation inhibition as observed in the ribosomal protein fraction (Fig. 1A). Symbols denote the number of generations of adaptation; colored symbols denotes glucose minimal media with increasing concentrations of chloramphenicol, grey symbols are strains grown in various nutrient conditions (Supplementary Data 2). Solid lines are the linear best-fit to the data (Supplementary Data 2). **B**. The ribosome-affiliated protein fraction **R** is proportional to the RNA/protein ratio ***r***, where **R**=0.67 x **r**. **C**. The increase in translation-limited intercept is a generic feature that does not depend upon the carbon source. Here, the black and purple symbols are as in panel B; the grey and lavender correspond to translation-limited growth in glycerol minimal media (Supplementary Data 2). Error bars denote mean squared error over at least 2 experiments done on different days.

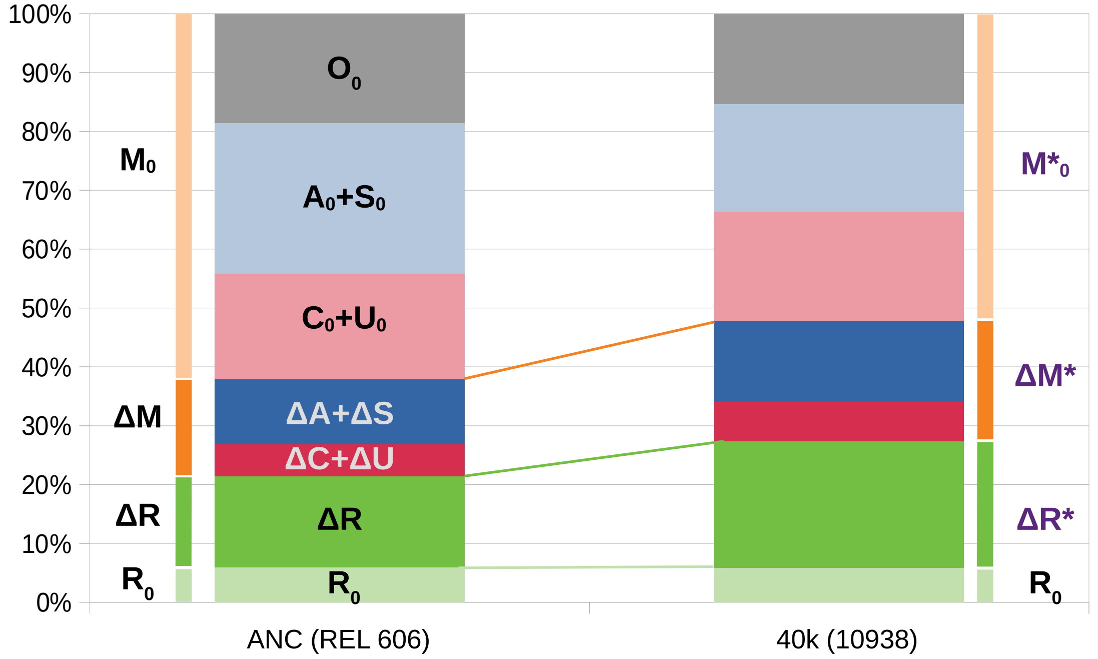

Figure S3 – Growth-dependent and growth-independent proteome fractions suing a 6-sector partitioning: The proteome allocation in glucose minimal medium for the ancestral (REL606) and 40k strain (10938) using the more detailed taxonomy proposed by Hui *et al.*[2] (described in Fig 2A of the main text) recapitulates the allocation inferred from the ribosome abundance shown in Fig. 1B of the main text. The growth-dependent fractions of the proteome increases from 30% in the ancestral strain to about 40% in the adapted strain, accommodated primarily by a decrease in the growth-rate independent fraction of the A sector and a decrease in the non-flux carrying O sector. The bars on the far-left and far-right correspond to the taxonomy in Fig. 1B. As in Fig. 1B, the growth-rate dependent fractions of ribosomal (**ΔR**, green) and metabolic (**ΔM**, orange) proteins increase concomitantly with the increased growth rate over the course of adaptation, at the expense of a diminished growth-rate independent fraction of metabolic proteins (**M0**, pale orange). Sectors with similar behavior (C/U and A/S) have been lumped together to improve the readability of the figure.

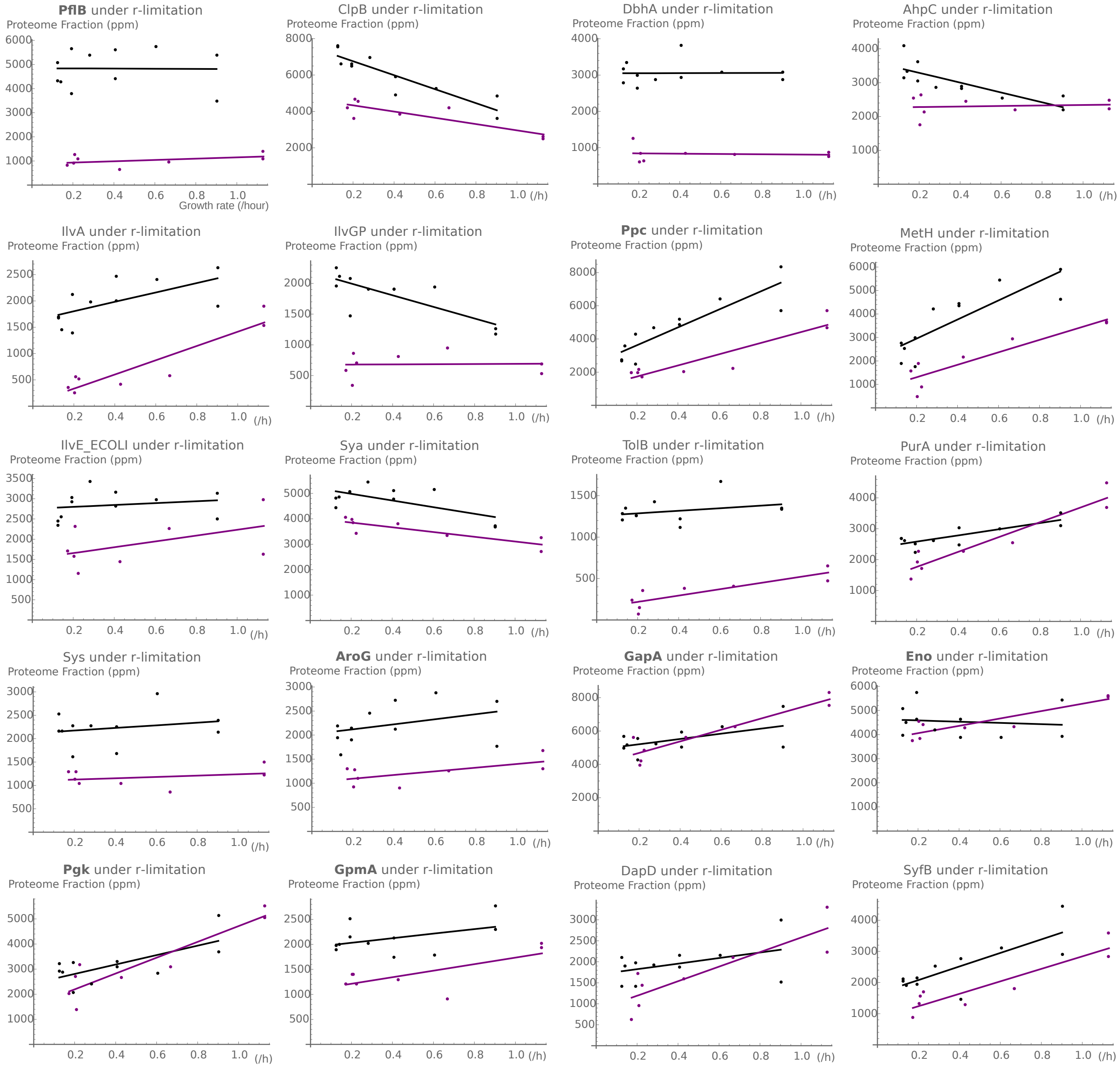

Figure S4 – Translation-limited protein fractions for individual proteins: Proteome fractions of individual S- and A-sector proteins under translation-limitation in the ancestral strain (REL606, black) and the 40k strain (10938, purple) as a function of the exponential growth rate. The proteins correspond to the entries of Fig. 3B in the main text.

Supplementary Notes

1. Linear fits to the proteomic data

Following previous work, we assign no growth dependence to the O-sector[2]; in all fit scenarios, the O-sector abundance is given by the average over all growth rates. The remaining proteomic data for each sector exhibits linear growth dependence under translation limitation. Below, we compare three fit scenarios using an increasing number of fitting parameters (Fig. S5).

The A-sector fit exhibits the largest variability among fitting scenarios. The highly-constrained fit shown in the main text (scenario 1) provides an accurate representation of the data despite the constraints (and the resulting small number of fit parameters).

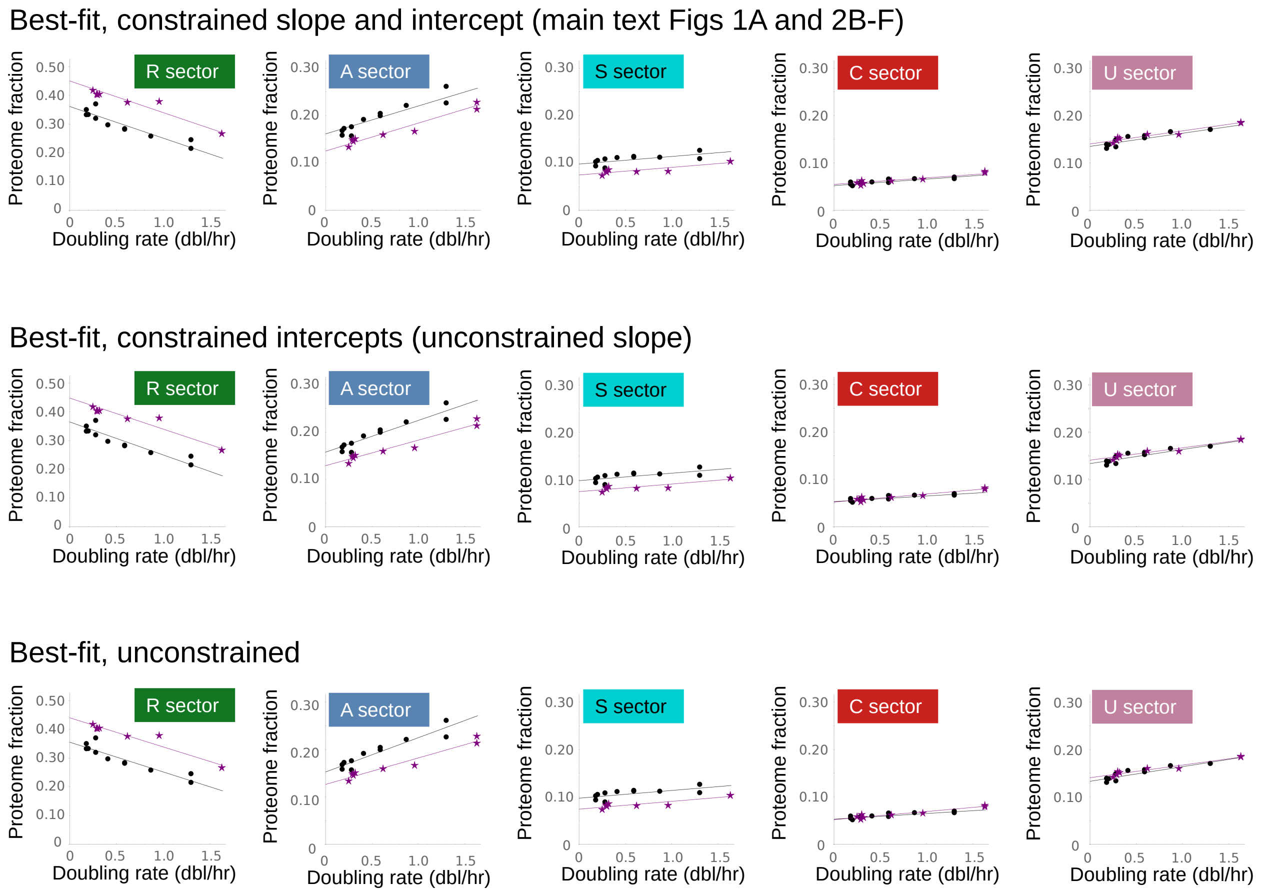

Figure S5 – Linear fit to the translation-limited protein fraction in the 6-sector model: Comparison of linear fits to the translation-limited proteomic data (black: Ancestral, purple: 40k). **Top**: Linear regression constrained so that the intercepts sum to 1 and the slope is identical between the ancestral and 40k strains [13 fitting parameters] (also shown in the main text, Figs. 1 and 2). **Middle**: Linear regression constrained so that the intercepts sum to 1 [18 fitting parameters]. **Bottom**: Fully-unconstrained linear regression [20 fitting parameters].

	R			A			S			C			U	
**Constrained slope, constrained intercept (main text Fig. 1A, Figs. 2B-F)**														
	Anc	40k		Anc	40k		Anc	40k		Anc	40k		Anc	40k
Slope (h/dbl)	-0.103			0.072			0.016			0.012			0.031	
Intercept	0.356	0.454		0.163	0.114		0.098	0.075		0.052	0.055		0.135	0.140
r^2	.846	.931		.856	.941		.473	.808		.747	.875		.815	.937
**Constrained intercept, unconstrained slope**														
	Anc	40k		Anc	40k		Anc	40k		Anc	40k		Anc	40k
Slope (h/dbl)	-0.115	-0.109		0.067	0.054		0.016	0.016		0.012	0.017		0.030	0.026
Intercept	0.365	0.448		0.157	0.129		0.098	0.075		0.053	0.052		0.134	0.141
r^2	.836	.936		.880	.942		.472	.808		.763	.914		.823	.938
**Unconstrained fit**														
	Anc	40k		Anc	40k		Anc	40k		Anc	40k		Anc	40k
Slope (h/dbl)	-0.103	-0.101		0.072	0.056		0.016	0.016		0.012	0.017		0.030	0.027
Intercept	0.356	0.441		0.153	0.126		0.097	0.074		0.053	0.052		0.133	0.141
r^2	.852	.942		.887	.944		.473	.808		.764	.914		.824	.938

Table S1 - Details of the linear regression shown in Fig. S5: The A-sector 40k slope exhibits the largest change among fit scenarios, although the r^2 is negligibly improved in the less-constrained fits.

2. Toy model of proteome response to flux-sensor removal

To illustrate the plausibility of the claim that the removal of a flux sensor is sufficient to produce a reduction in the translation-limited intercept of upstream enzymes, we have constructed a minimal toy model that contains four important features.

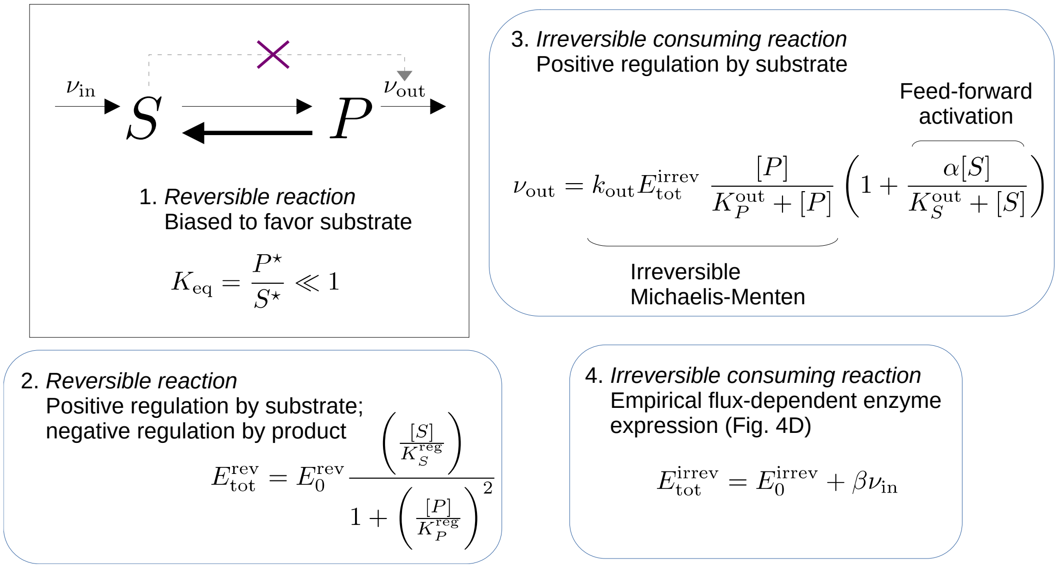

Figure S6 - Details of the toy model shown in Fig. 5 of the main text: The purple cross denotes abrogation of the flux-sensing mechanism after adaptation.

Important features of the toy model:

1 The substrate concentration $[S]$ is directly correlated with the in-flux $\nu_{\rm in}$ if the reversible substrate-consuming reaction is strongly biased in the reverse direction[8].

2 The total enzyme abundance of the reversible substrate-consuming reaction is positively-regulated by the substrate concentration $[S]$ and negatively regulated by the product concentration $[P]$.

3 The out-flux $\nu_{\rm out}$ of product is mediated by a set of irreversible enzymes, some of which are under positive feed-forward activation (FFA) by the substrate concentration $[S]$ (or, equivalently, by a transcription factor that is activated by the substrate). The factor α is our control parameter for de-activating the flux-sensor.

4 To close the system, the enzymes mediating the consumption reaction, $E_{\rm tot}^{\rm rev}$, exhibit the linear positive correlation with the influx that is observed experimentally when growth-rate (assumed to be proportional to the in-flux $\nu_{\rm in}$) is modulated by translation inhibition.

Written as a set of differential equation for the concentrations of substrate $[S]$ and product $[P]$,

$$\frac{dS}{dt} = \nu_{\rm in} - k_{\rm rev} E_{\rm tot}^{\rm rev} \left(\frac{[S] - \frac{[P]}{K_{\rm eq}}}{K_{\rm S} + [S] + \frac{[P]}{K_{\rm P}}} \right),$$

$$\frac{dP}{dt} = k_{\rm rev} E_{\rm tot}^{\rm rev} \left(\frac{[S] - \frac{[P]}{K_{\rm eq}}}{K_{\rm S} + [S] + \frac{[P]}{K_{\rm P}}} \right) - \nu_{\rm out}, \tag{S1}$$

where we have used the following parameter values to illustrate the behavior of the system:

**Toy model parameters**			
k_{rev}	5	E_0^{rev}	0.001
k_{out}	5	K_{eq}	0.01
$K_{\text{P}}^{\text{reg}}$	0.025	E_0^{irrev}	0.01
K_{P}	1	β	0.2
$K_{\text{P}}^{\text{out}}$	0.1		
$K_{\text{S}}^{\text{reg}}$	0.025	α	$\begin{cases} \alpha_{\text{max}} = 2 \text{ (FFA)} \\ \alpha_{\text{min}} = 0 \text{ (no FFA)} \end{cases}$
K_{S}	0.05		
$K_{\text{S}}^{\text{out}}$	0.1		

Table S2 - Parameter values used in the toy model to generate the output shown in Fig. 5 of the main text: These parameters are purposely unit-less to emphasize that the toy model illustrates the plausibility that loss of a flux-sensing mechanism can create space in the proteome; the toy model is not intended as a detailed mechanistic model.

Deletion of the enzyme responsive to flux-sensing (as in the case of PykF in the Ara-1 lineage described in the main text) corresponds to setting the feed-forward-activation parameter $\alpha_{\text{min}} = 0$; partial loss-of-function corresponds to $\alpha_{\text{max}} > \alpha_{\text{min}} > 0$, where the smaller the change in α, the smaller the change in the zero-flux intercept for the total enzyme abundance $E_{\text{tot}}^{\text{rev}}$.

Both the flux-sensing capability of the substrate and the drop in the total enzyme abundance $E_{\text{tot}}^{\text{rev}}$ rely upon strong bias of the reversible reaction in the direction of the substrate, $K_{\text{eq}} \ll 1$. Reduction of this thermodynamic bias decreases the sensitivity of the substrate concentration to the in-flux ν_{in} and decreases the change in the zero-flux intercept for the total enzyme abundance $E_{\text{tot}}^{\text{rev}}$ when flux-sensitivity is lost in the consuming reaction ($\alpha \to \alpha_{\text{min}}$).

References

1. Barrick, J. E. *et al.* Genome evolution and adaptation in a long-term experiment with Escherichia coli. *Nature* **461**, 1243–1247 (2009).
2. Hui, S. *et al.* Quantitative proteomic analysis reveals a simple strategy of global resource allocation in bacteria. *Mol. Syst. Biol.* **11**, 784 (2015).
3. Neidhardt, F. C., Bloch, P. L. & Smith, D. F. Culture Medium for Enterobacteria. *J. Bacteriol.* **119**, 736–747 (1974).
4. Greulich, P., Scott, M., Evans, M. R. & Allen, R. J. Growth-dependent bacterial susceptibility to ribosome-targeting antibiotics. *Mol. Syst. Biol.* **11**, 796 (2015).
5. Herbert, D., Phipps, P. J. & Strange, R. E. Chapter III Chemical Analysis of Microbial Cells. in *Methods in Microbiology* vol. 5 209–344 (Elsevier, 1971).
6. Benthin, S., Nielsen, J. & Villadsen, J. A simple and reliable method for the determination of cellular RNA content. *Biotechnol. Tech.* **5**, 39–42 (1991).
7. Sperling, E., Bunner, A. E., Sykes, M. T. & Williamson, J. R. Quantitative Analysis of Isotope Distributions In Proteomic Mass Spectrometry Using Least-Squares Fourier Transform Convolution. *Anal. Chem.* **80**, 4906–4917 (2008).
8. Euler, C. & Mahadevan, R. On the design principles of metabolic flux sensing. *Biophys. J.* **121**, 237–247 (2022).
